# Physical activity promotion in the primary care setting in pre- and type 2 diabetes - the Sophia step study, an RCT

**DOI:** 10.1186/s12889-015-1941-9

**Published:** 2015-07-12

**Authors:** Jenny Rossen, Agneta Yngve, Maria Hagströmer, Kerstin Brismar, Barbara E. Ainsworth, Christina Iskull, Peter Möller, Unn-Britt Johansson

**Affiliations:** Sophiahemmet University, Stockholm, Sweden; Department of Clinical Sciences and Education, Södersjukhuset, Karolinska Institutet, Stockholm, Sweden; School of Hospitality, Culinary Arts and Meal Sciences, Örebro University, Örebro, Sweden; Department of Neurobiology, Care Sciences and Society, Division of Physiotherapy, Karolinska Institutet, and Department of Physical Therapy, Karolinska University Hospital, Stockholm, Sweden; Department of Molecular Medicine and Surgery, Karolinska Institutet, and Rolf Luft Research Center for Diabetes and Endocrinology, Karolinska University Hospital, Stockholm, Sweden; School of Nutrition and Health Promotion, Arizona State University, Phoenix, AZ USA; Sophiahemmet Hospital, Stockholm, Sweden

**Keywords:** Metabolic health, Pedometer, Adults, HbA_1c_, Behavior change, Intervention

## Abstract

**Background:**

Physical activity prevents or delays progression of impaired glucose tolerance in high-risk individuals. Physical activity promotion should serve as a basis in diabetes care. It is necessary to develop and evaluate health-promoting methods that are feasible as well as cost-effective within diabetes care. The aim of Sophia Step Study is to evaluate the impact of a multi-component and a single component physical activity intervention aiming at improving HbA_1c_ (primary outcome) and other metabolic and cardiovascular risk factors, physical activity levels and overall health in patients with pre- and type 2 diabetes.

**Methods/design:**

Sophia Step Study is a randomized controlled trial and participants are randomly assigned to either a multi-component intervention group (A), a pedometer group (B) or a control group (C). In total, 310 patients will be included and followed for 24 months. Group A participants are offered pedometers and a website to register steps, physical activity on prescription with yearly follow-ups, motivational interviewing (10 occasions) and group consultations (including walks, 12 occasions). Group B participants are offered pedometers and a website to register steps. Group C are offered usual care. The theoretical framework underpinning the interventions is the Health Belief Model, the Stages of Change Model, and the Social Cognitive Theory. Both the multi-component intervention (group A) and the pedometer intervention (group B) are using several techniques for behavior change such as self-monitoring, goal setting, feedback and relapse prevention.

Measurements are made at week 0, 8, 12, 16, month 6, 9, 12, 18 and 24, including metabolic and cardiovascular biomarkers (HbA_1c_ as primary health outcome), accelerometry and daily steps. Furthermore, questionnaires were used to evaluate dietary intake, physical activity, perceived ability to perform physical activity, perceived support for being active, quality of life, anxiety, depression, well-being, perceived treatment, perceived stress and diabetes self- efficacy.

**Discussion:**

This study will show if a multi-component intervention using pedometers with group- and individual consultations is more effective than a single- component intervention using pedometers alone, in increasing physical activity and improving HbA_1c_, other metabolic and cardiovascular risk factors, physical activity levels and overall health in patients with pre- and type 2 diabetes.

**Trial registration:**

ClinicalTrials.gov Identifier: NCT02374788. Registered 28 January 2015.

## Background

Having type 2 diabetes, but also pre-diabetes increases the risk of cardiovascular disease (CVD) and premature death [1]. Physical activity on a regular basis enhances metabolic control [2, 3], improves blood lipid profile, blood pressure and quality of life [2, 4]. Several studies have shown preventive effects of physical activity in individuals with impaired glucose tolerance [5–9] and physical activity in persons with type 2 diabetes clearly lowers the risk of cardiovascular disease and premature death [10, 11]

The recently updated Swedish recommendations for physical activity for persons with type 2 diabetes are in line with the US PA Guidelines recommendation of 2008 [12, 13]. The recommendations are “To undertake at least 150 min per week of moderate to vigorous intensity aerobic physical activity spread out during at least 3 days during the week with no more than two consecutive days between the bouts and moderate to vigorous resistance training at least 2-3 days per week [2, 12]. There is a dose–response relationship between aerobic physical activity and health gains, and activity duration beyond 150 min per week is associated with an even greater decline in HbA_1c_ [14] as well as a reduced risk of cardiovascular disease and all-cause mortality in patients with diabetes [10]. An increased body of evidence is pointing at the importance of resistance training or combined training for blood glucose control [2, 3, 15] and a high relative muscle mass for better insulin sensitivity [16]. It is also evident that reducing sedentary time and breaking up sitting time may give additional health benefits over the recommended activities and might be of importance especially for the most inactive and unfit individuals [2, 17, 18]. The most important component to maintain these beneficial effects seems to be continuous repetition and it is therefore crucial that physical activity is perceived as enjoyable and that it is incorporated in daily routines [2]. In the Swedish population, 52 % of adults are regarded as being sufficiently active [19] and self-reported data from the Swedish National Diabetes Register showed that in the population with diabetes 55 % reached 3 × 30 min per week [11]. It is considered crucial to find and evaluate strategies to increase the adoption and maintenance of regular physical activity in the population with pre-and type 2 diabetes [2, 11].

Advising physical activity is both cost-effective and feasible in primary care [20, 21] and promotion of physical activity should serve as a basis in diabetes care [1, 22]. The Board of Health and Welfare (Socialstyrelsen) in Sweden gives high priority to physical activity in diabetes care and since 1999 strongly recommends primary care to provide advice and support for individuals with diabetes as well as for individuals with increased risk for developing diabetes [23, 24]. Given the low number of people with diabetes being sufficiently active, the promotion of physical activity in the Swedish diabetes care is not satisfactorily effective today and needs to be improved [25].

In the literature, a number of methods reinforcing physical activity promotion in primary care have been evaluated and shown to be effective. The World Health Organization put emphasis on primary care to encourage and support people to take better care of their own health and to use evaluated tools and technology for this purpose [26]. Pedometers have been helpful in increasing physical activity levels and in improving metabolic parameters in patients with diabetes in several previous studies [27–29]. Pedometers have also been efficient in increasing the number of steps with a subsequent improvement in blood pressure even in already healthy individuals [30]. An advantage with pedometers is their efficiency in increasing the motivation to be more active and less sedentary on a daily basis, which is especially important for metabolic control and in lowering blood pressure. Well-planned group counseling sessions led by health professionals have also been shown to be effective in reducing diabetes risk in several previous interventions [28, 31–33]. The group setting offers social support and a pronounced opportunity for participants to share experiences, encouragement and to strengthen change. Group education is emphasized in the Swedish diabetes care [23, 24] but is today used by only 24 % of primary care units [25].

Recent reviews on physical activity interventions in primary care demonstrate that exercise prescription is a successful method to increase physical activity up to 12 months [21, 34]. In the Swedish primary care, a treatment method named FaR® (abbreviation for Physical Activity on Prescription) is recommended for use in a number of diseases including type 2 diabetes [35]. The focus is on person-centered counseling and the current diagnosis, health status, history of physical activity, risks and preferences of the individual serves as a basis for the counseling. FaR is based on Social Cognitive Theory and the Stages of Change Model. In an evaluation 2010 87 % of the Swedish health care centers had implemented FaR [36].

Motivational Interviewing is a counseling method using a person-centered approach [37]. There is some inconsistency in the evidence for motivational interviewing both in diabetes care and in increasing physical activity in the primary care setting: unfortunately many studies fail to pick up treatment fidelity, the qualification of the professionals delivering the treatment, mode of delivery and intervention intensity [38, 39]. Alongside with other intervention components, such as self-monitoring, physical activity on prescription and offering more than two motivational interviewing sessions the efficacy may be improved [39]. In the promotion of physical activity flexibility using various approaches is recommended [40–42] as well as to tailor the intervention to the individual [43].

There is still a gap in the evidence on which methods, what intervention components and what support intensity would be most effective in increasing long-term physical activity in in the primary care setting for persons with pre- and type 2 diabetes [21, 43, 44]. More evidence is also needed on maintenance strategies for long-term effectiveness [33, 44].

Based on these identified gaps in knowledge, Sophia Step Study has been developed as an evidence-based structured, two-year health promotion program at two intensity levels of support focusing on physical activity and aimed for the primary care setting. The study aims to evaluate and explore the impact of two levels of intervention intensities of physical activity support on health parameters.

The aim of this paper is to describe the design and recruitment procedure, methods, and the theoretical framework for the physical activity promotion program Sophia Step Study.

## Methods/design

### Main objective

The main objective of Sophia Step Study is to evaluate the impact of a multi- component and a single-component primary care physical activity intervention aiming at improving HbA_1c_ (primary outcome) and other metabolic and cardiovascular risk factors, physical activity levels and overall health in patients with pre-diabetes and type 2 diabetes.

### Hypothesis

The hypothesis is that both levels of intervention have effect on the primary outcome HbA_1c_, with the multi-component intervention having superior and longer lasting effects.

### Study design and recruitment

The Sophia Step Study is a two-year randomized controlled trial (RCT) with three parallel groups. The CONSORT statement is followed [45]. The study takes place at the primary health care centers at Sophiahemmet, Stockholm, Sweden.

**Fig. 1:**
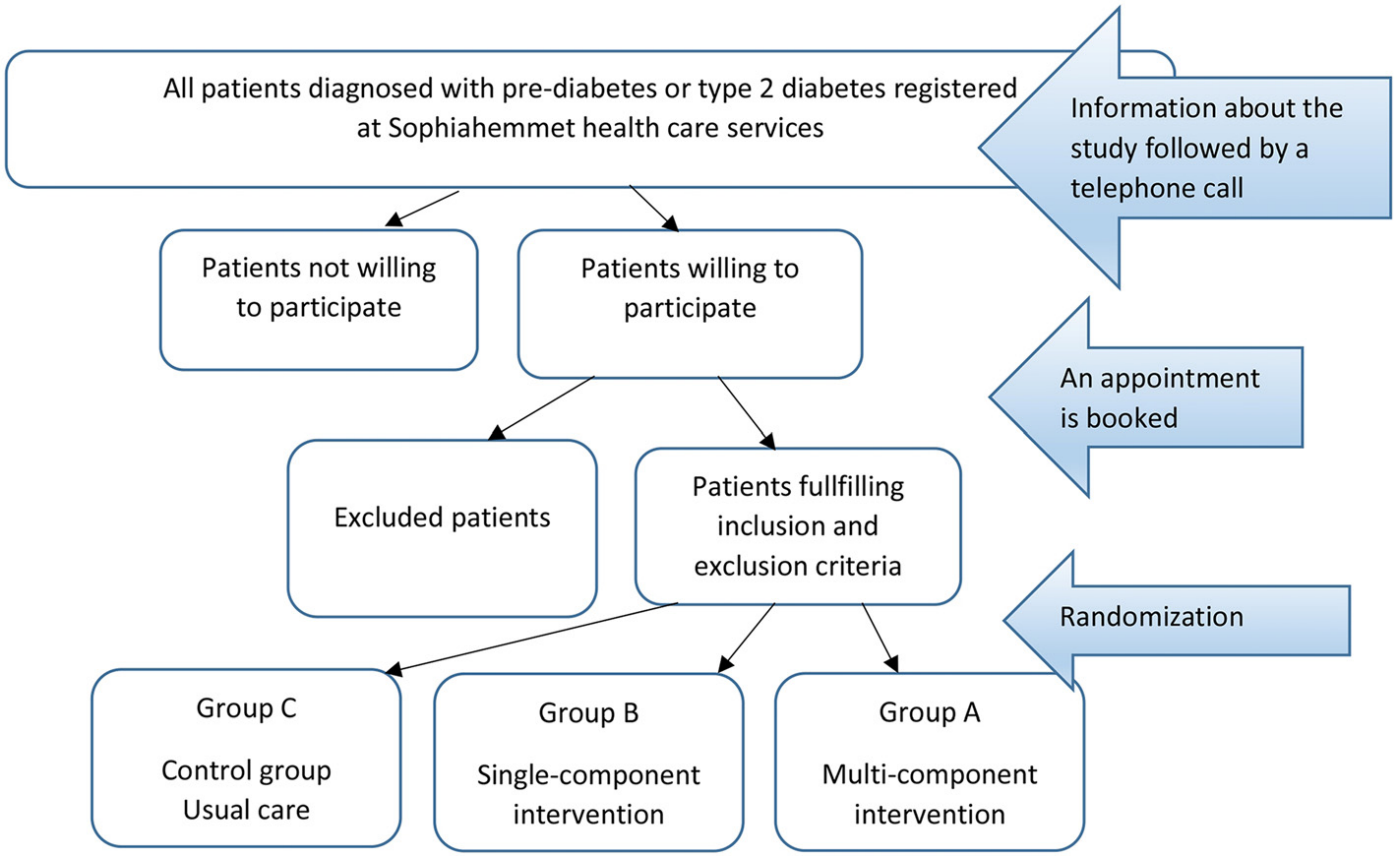
Recruitment procedure and randomization of Sophia Step Study subjects

All patients at the health care centers diagnosed with pre-diabetes and type 2 diabetes and fulfilling the inclusion criteria are informed of the study and asked whether they are interested in participating (Fig. 1). Patients showing interest receive a letter with further information and are subsequently interviewed over telephone by the diabetes specialist nurse and asked a set of inclusion/exclusion questions. If they fulfil the inclusion criteria they are booked for a baseline control.

#### Inclusion criteria

Age 40-80 years and ability to communicate in Swedish. Either Pre-diabetes (HbA_1c_ > 39- < 47 mmol/mol and/or fasting glucose >5.6 mmol/l) or diagnosed with type 2 diabetes with a duration of ≥1 year.

#### Exclusion criteria

Myocardial infarction in the past 6 months, serum creatinine >140 mmol/l, diabetic foot ulcer or risk of ulcer (severe peripheral neuropathy), on insulin since the last 6 months, additional disease prohibiting physical activity, repeated hypoglycemia or severe hypoglycemia in the past 12 months, being very physically active according to the Stanford Brief Activity Survey [46] or having no access to internet.

### Procedure and timeline

**Fig. 2:**
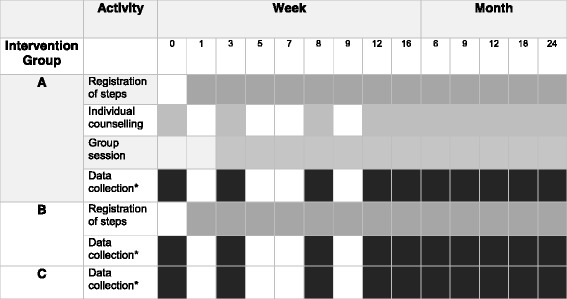
Time frame for Sophia Step Study. Time points for intervention components (grey) and data collection (black) for all groups. *Including blood samples, anthropometric and physical activity measurements and questionnaires. More details are depicted in Table 1

The baseline measurements start with an examination by the patient’s general practitioner for exclusion purposes. A notification is made on subjects not fulfilling the criteria at this stage. The study-specific measurements are made by the diabetes specialist nurse or a trained assistant, followed by randomization to three groups using closed envelopes while stratified by gender. The participants are randomly assigned to either the multi-component intervention group (A), the single component group (B) or a control group (C). A total of 310 patients with pre- diabetes or type 2 diabetes will be included gradually, with the aim of 100 participants included in group A and B, and 110 in the control group C by the end of 2017. After randomization, participants are given a schedule for measurement time points and group A participants receive a schedule for group sessions and individual consultations. The intervention lasts for 24 months, with more intensive support from the health care professionals within the first 24 weeks, and less support in the second year (Fig. 2).

Sophia Step Study started with a pilot group in March 2013, entailing 8 participants in group A and 6 in group C. The pilot revealed compliance to the protocol and ability to recruit and the first participants were recruited in November 2013. The plan is to have 310 participants completed the intervention in 2020.

### Intervention

#### Pedometer

In week 1, participants in the intensive intervention group (A) and the pedometer group (B) arrive for their second visit at the health care center. They are offered a pedometer (YAMAX; model Yamax Digiwalker SW 200: Yamax Corporation, Tokyo, Japan), instructions for how to use the pedometer, how to record their daily steps and to set a daily step goal on a website (www.steg.se, Select Wellness AB, Stockholm, Sweden). The participants register steps daily in a diary, and are recommended to enter them onto the website weekly. Non-ambulant activities such as biking and swimming are translated into steps by a simple calculation (each 30 min of activity, regardless of intensity, gives 3500 steps). On the website a “healthy goal” of minimum 7000 step per day is depicted as a reference [47]. The participants are asked to decide on individual goals after the first week of wearing the pedometer. If a pedometer stops working or gets lost the participants are encouraged to pick up a new pedometer at the health care center or a new pedometer is sent by post.

#### Group counseling

**Fig. 3:**
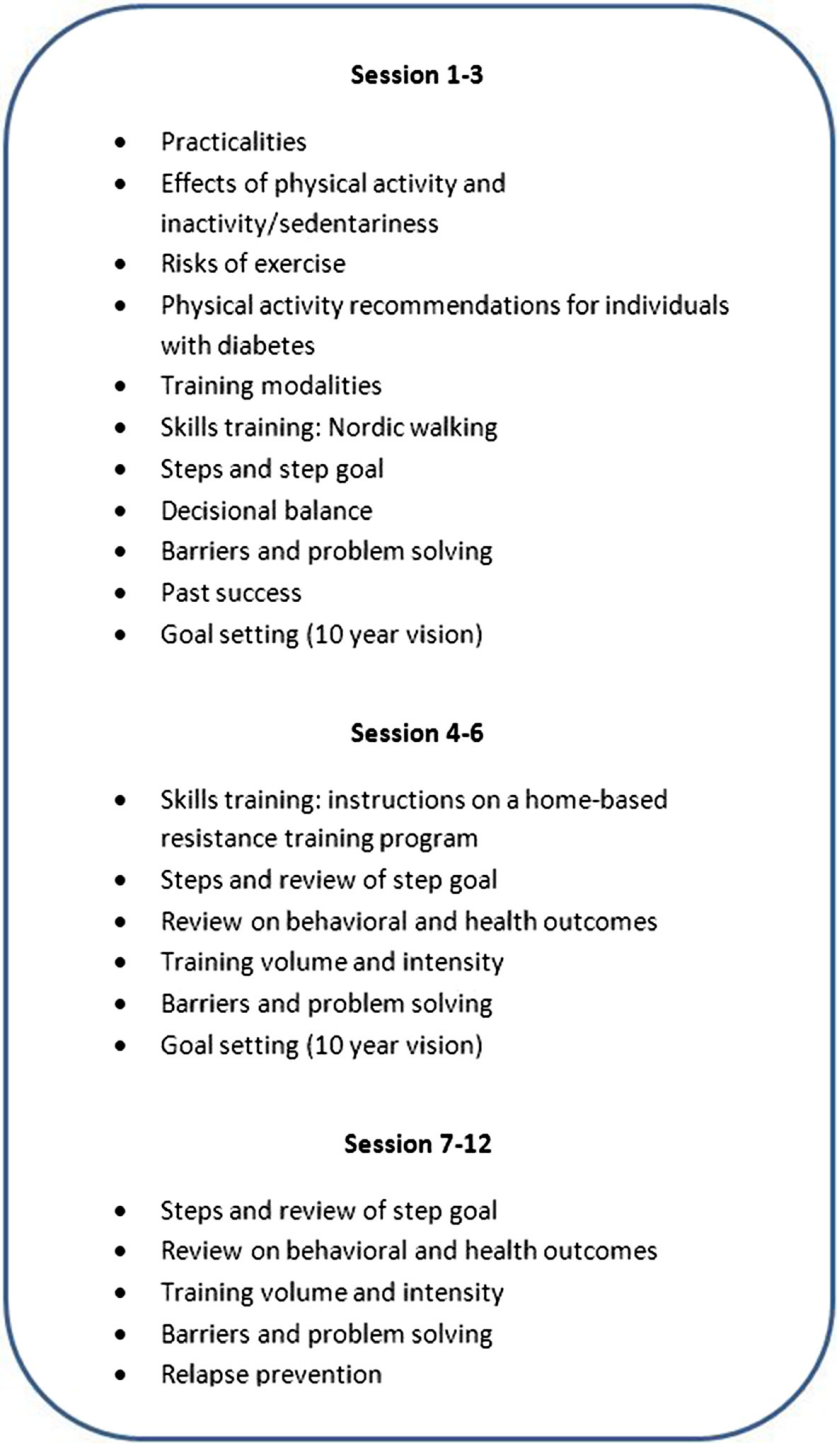
Group counseling program for group A with the content and the behavior change techniques

Group A participants are offered 12 group meetings (Fig. 2) over two years’ time, with the majority of meetings being held in the first six months. The group meetings include a 30 min walk and 60 min group consulting and are steered by a health professional trained in physical activity promotion and familiar with models and techniques for behavior change. A workbook developed for the project, based on the Health Belief Model, the Stages of Change Model and Social Cognitive Theory is used. The content of the group counseling program and the behavior change techniques used is shown in Fig. 3. The order of the content may shift depending on holidays, season or other concerns that arise.

#### Person-centered individual counseling

Group A participants are offered individual consultations with their diabetes specialist nurse at 9 occasions. The nurses are trained in MI and are using an MI-spirit in their consultations. The consultation takes place concurrently with the study measurements and the health outcomes from the measurements and the number of steps taken recently serves as a basis for these talks. Moreover, the nurse is, during one of the first MI-talks prescribing physical activity according to the method FaR [35]. The nurse also informs participants about the opportunities that follow a prescription, such as subsidies to gyms and sport clubs and trained staff at certain sport clubs. The prescription is followed up yearly. All in all, the meetings take 45-60 min each.

#### Usual care

The control group (C) receives diabetes care as usual, except for the extra measurements included in the study. Usual care consists of seeing a diabetes specialist nurse and a general practitioner at least once a year and receiving lifestyle advice, including advice on physical activity. Depending on the metabolic status of the patient there might be consultations with both the nurse and/or the general practitioner more often. The number of consultations made will be recorded for each participant. A physical activity prescription might also be issued as a part of usual care, but not in a systematic way.

### Theoretical framework

It is widely recommended that program design should be based on a theory and the behavior change techniques used should be depicted to improve evidence synthesis [21, 43, 48]. The theoretical framework underpinning the two interventions in Sophia Step Study is the Health Belief Model, the Stages of Change Model and the Social Cognitive Theory [49]. Both the multi-component intervention (group A) and the pedometer intervention (group B) are using several behavior change techniques based on the CALO-RE taxonomy [48].

**Fig. 4:**
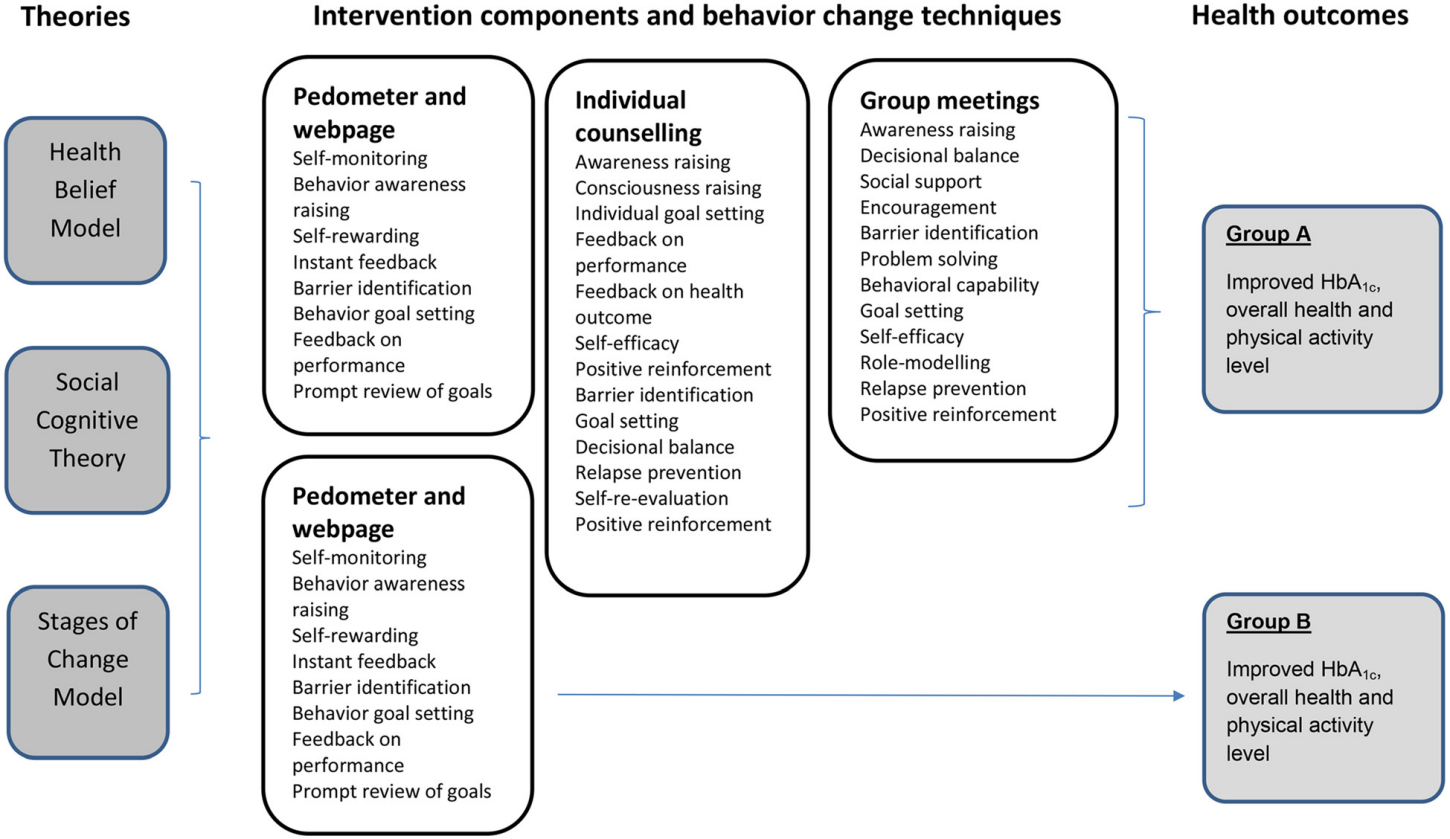
Conceptual framework of Sophia Step Study. The conceptual framework depicts the underlying theories, the intervention components with the behavior change techniques used and the expected outcomes for the two intervention groups

Based on these theories various intervention components were chosen to offer flexibility; to adjust to individual differences and preferences and to strengthen change. A conceptual framework visualizing the intervention program is depicted in Fig. 4.

### Outcome measures

Measurements are made on all participants at week 0, 8, 12, 16, 24, month 9, 12, 18 and 24. The primary outcome variable is HbA_1c_. Health outcomes and measurement time points are summarized in Table 1. The measurements are planned to be performed within ± 2 weeks and notes are made if they are made sooner or later than this.

**Table 1:** Outcome parameters and time points for measurements for all groups in Sophia Step Study

Variables	Baseline	Trial	Trial	Trial	Trial	Trial	Trial	Trial	Trial
		w. 8	w. 12	w. 16	6 m	9 m	12 m	18 m	24 m
**Biomarkers**									
HbA_1c_ (mmol/mol)	✓		✓		✓		✓		✓
Plasmaglucose (fasting) mmol/l	✓		✓		✓		✓		✓
Triglycerides (mmol/l)	✓		✓		✓		✓		✓
LDL (mmol/l)	✓		✓		✓		✓		✓
HDL (mmol/l)	✓		✓		✓		✓		✓
Total Cholesterol (mmol/l)	✓		✓		✓		✓		✓
Free fatty acids (mmol/l)	✓		✓		✓		✓		✓
Insulin (mU/l)	✓		✓		✓		✓		✓
IGF BP1 (μ/l)	✓		✓		✓		✓		✓
ApoA1 (g/l)	✓		✓		✓		✓		✓
ApoB (g/l	✓		✓		✓		✓		✓
C-peptid (pmol/l)	✓		✓		✓		✓		✓
**Anthropometry**									
Weight (kg)	✓	✓	✓	✓	✓	✓	✓	✓	✓
% Body fat	✓	✓	✓	✓	✓	✓		✓	✓
Height (cm)	✓						✓		
BMI (kg/m^2)^	✓	✓	✓	✓	✓	✓	✓	✓	✓
Waist circumference (cm)	✓	✓	✓	✓	✓	✓	✓	✓	✓
Sagittal Abdominal Diameter	✓		✓		✓	✓	✓		✓
Resting pulse and blood pressure	✓	✓	✓	✓	✓	✓	✓	✓	✓
**Physical activity**									
Physical activity level (counts/min)	✓				✓		✓	✓	✓
Resistance training									
Resistance training and hand grip strenght	✓		✓		✓	✓	✓	✓	✓
**Questionnaires & Health Measures**									
Demographic data	✓								
Smoking and snuffing habits	✓								✓
Dietary habits, FFQ	✓						✓		✓
Drinking habits	✓				✓		✓		✓
Stress and working conditions	✓		✓		✓		✓		✓
EQ-5D 3L	✓		✓		✓		✓		✓
Overall health and sleep	✓		✓		✓		✓		✓
IPAQ-short	✓		✓		✓		✓		✓
Social support for exercise	✓		✓		✓		✓		✓
Self-efficacy for exercise	✓		✓		✓		✓		✓
Neighborhood environment	✓		✓		✓		✓		✓
HADS	✓		✓		✓		✓		✓
PSS	✓		✓		✓		✓		✓
Swe-PAID-20	✓		✓		✓		✓		✓

#### Biomarkers

Measurements include fasting blood samples on HbA_1c_ (mmol/mol), plasma glucose (mmol/l), triglycerides (mmol/l), LDL (mmol/l), HDL (mmol/), total Cholesterol (mmol/l), free fatty acids (mmol/l), Insulin (mU/l), IGF BP1 (μ/l), Apolipoprotein-A1 (g/l), Apolipoprotein B (g/l) and C-peptid (nmol/l). HbA1c, haemoglobin A1c (ref < 5.2 %) is determined with immunologic MonoS method, Unimate (Roche Diagnostics, Basel, Schweiz). To convert HbA1c MonoS into HbA1c (DCCT) NGSP (National Glycoprotein Standardization Programme) the formula, NGSP = 0.92*MonoS + 1,33 is used. Plasma glucose are determinedwith a glucose oxidase method, total Cholesterol and triglycerides are determined by using enzymatic method, LDL and HDL are determined by using a homogeneous method, Apolipoprotein-A1 and Apolipoprotein B are determined by using turbimetric method and C-peptid are determined by using immunometric method using two monoclonal antibodies and detection with electrochemiluminiscense using a Modular E system (Beckman Coulter, Inc.). Serum insulin concentrations are determined with RIA-kits purchased from Pharmacia & Upjohn, Stockholm. IGFBP-1 concentrations in serum are determined by RIA according to the method of Póvoa et al. [50]. Samples are saved for later free fatty acid analyses.

#### Anthropometry

Anthropometric measurements include weight and percentage body fat using Tanita digital scale (Model TBF-300A, Arlington Heights, IL). Weight is measured with light clothes, no shoes to the nearest 0.1 kg. Height is measured at the first visit by use of a calibrated stadiometer to the nearest centimeter. Waist circumference is measured with SECA 201 tape, horizontal around the waist 2 cm above the umbilicus. Sagittal abdominal diameter is measured with the subject in a supine position with the knees expanded at the level of the umbilicus using a Holtain-Kahn abdominal caliper (Holtain, Ltd., Crosswell, Crymych; Dyfed, UK). Resting pulse and blood pressure is measured with Omron M6 Comfort.

#### Objectively measured physical activity

To objectively assess total physical activity as well as sedentary time and time spent in different intensities the ActiGraph GT1M accelerometer (ActiGraph, Pensacola, FL) will be used. The participants are asked to wear the accelerometer placed on the back all wakening hours for seven consecutive days. The accelerations are sampled at 10 Hz i.e. 10 times per second and data is summarized over one minute and outputted as numerical counts. Total physical activity is expressed as total counts and steps per day. Time spent sedentary and in different intensities is derived from established cut-points according to Freedson (1998) and Matthews (2005 and 2008) [51–53].

#### Resistance training

Initiation of regular resistance training is reported contemporary with the measurement. Hand grip strength is measured as a proxy for overall muscle strength [54]. Hand grip strength is measured in kilograms using the hand-held Saehan Hydraulic Hand Dynamometer, model SH5001 (former Jamar) (Saehan Corporation, Masan, South Korea).

#### Questionnaires

A web-site delivered questionnaire is e-mailed to the participants at baseline, week 12, and at 6, 12 and 24 months. The questionnaire takes 30-40 min to complete, incorporates several validated questionnaires and some study specific questions including demographic and lifestyle characteristics that aims to evaluate diet, physical activity, motivational circumstances to be physically active, overall health and well-being and problem areas in diabetes.

*Demographic data* are collected by study specific questionnaire with items on civil status, having children under 18, caring for relatives, educational level and income.

*Smoking and snuffing habits* are measured using questions on current and previous habits and the dose.

*An indication of dietary habits* is measured by a Food Frequency Questionnaire (FFQ) developed and validated by The Swedish National Food Agency. This questionnaire is recommended to use in Swedish populations for its validity and reproducibility and for comparable reasons [55].

*Drinking habits* are assessed by two items based on the amount and regularity of alcohol consumption [56].

*Stress and working conditions* are measured by 4 items on over-time, paid overtime, having subordinates and perceived work security.

*Health outcome* is measured using The EuroQol (EQ-5D 3L) that includes questions on mobility, hygiene, daily activities, pain/ discomfort and anxiety/depression. Within the particular EQ-5D dimension the responses are within three levels of severity; no problems, some or moderate problems and extreme problems. The questionnaire also measures overall health status on a vertical visual analogue scale where 0 indicates worst imaginable health and 100 best imaginable health [57]. An approval to use the instrument in the current project is received from The EuroQol group.

*Overall health and sleep* is measured by a 1-100 scale for health condition where 0 is worst possible and 100 is best possible. One question on difficulties falling asleep and one question on sleep quality.

*Subjectively assessed physical activity* is measured with The International Physical Activity Questionnaire (IPAQ), a self-administered 7-item questionnaire that evaluates the frequency and duration of walking, moderate- and vigorous-intensity physical intensity, and minutes spent sitting during the past week [58].

*Social support for exercise* is measured with Physical Activity Social Support (PASS) first developed by Sallis et al. [59]. The PASS examines general support (1 item), friend (2 items), family (2 items) and colleagues support for exercise. PASS is using a 4-point Likert response scale (1 = strongly agree to 4 = strongly disagree).

*Self-efficacy for exercise* is measured with the 5-item scale The Self-Efficacy for Exercise Scale. It assess one’s confidence to continue exercising when feeling tired, being in a bad mood, not having time, being on vacation and at bad weather [60].

*Neighborhood environment* is assessed by the scale Neighborhood Environment developed by Mujahid et al., 2007 and using a 5-point responses (1 = strongly agree to 5 = strongly disagree) [61]. In this study 17 items about abilities to undertake exercise and walking, availability of foods, safety and social environment are used.

*Depression and anxiety* is measured with The Hospital Anxiety and Depression Scale (HADS): HADS is a 14 item questionnaire consisting of two subscales; depression and anxiety, with seven items each. The items are graded on a four-point Likert scale ranging from 0-3. The total score ranges from 0-21 for the HADS depression scale and ranges from 0-21 for the HADS anxiety scale [62].

*Stress* is measured with The Perceived Stress Scale (PSS) which is a 14- item questionnaire used to measure how stressful different situations in one's life are perceived (Cohen et al. 1983). The items are graded on a five-point Likert scale. The total score for PSS ranges from 0-56. Eskin et al., 1996 have psychometrically tested the Swedish version of PSS [63].

*Diabetes distress* is measured using The Problem Areas in Diabetes questionnaire (Swe-PAID-20): This is a 20 items questionnaire translated in to Swedish by Amsberg et al. [64]. The patient rates their distress with having diabetes on a five-point Lickert scale, from 0 = not a problem to 4 = serious problem. The total score ranges from 0-100. The original version of PAID was developed by Polonsky et al., 1995 [65]. These questions are only answered by the participants diagnosed with diabetes.

### Fidelity criteria

Attendance and notes on reasons for absence at individual visits and group consultation is made after each session. Compliance to registration of steps is made monthly and reasons for failing to register steps is tracked and noted. Notes from the MI talk are registered in the health journal. The project group has regular meetings to discuss issues regarding MI talks, physical activity on prescription and measurements, in order to assure the routines of both measurements and intervention components. The quality of each individual counseling session is assessed on a 1-10 scale by the diabetes specialist nurse for both motivational interviewing and physical prescription. A qualitative study to explore how the participants perceived the support as well as barriers and facilitators will help to evaluate the intervention efficacy from a patient perspective.

### Sample size and planned statistical analysis

The sample size calculation assumed 80 % power and was calculated drawing on previous literature [14, 66] by statistical power analysis. To detect a difference of > 0.6 mmol/mol in HbA_1c_ with a standard deviation of 1.2 % at 12 months between group A and B and between group A and C we need 56 per group. Taking account the compliance and drop-out (30 %) and patients which decline participation (20 %) we need in group A 100 patients, in group B 100 patients and in group C 110 patients.

The study will be evaluated with both quantitative and qualitative methods and a cost-effectiveness analysis will be performed. The CONSORT 2010 statement will be used for description and analysis [45]. Data will be analyzed following the Intent-to-treat approach (ITT). Descriptive statistics will be used to describe the study population at baseline. The study data will be examined for outliers, normality and missing data. Potential confounders as (e.g. BMI at baseline, gender and age) will be used if there are differences at baseline.

Un-paired and paired tests, correlation coefficients as well as ANOVAs will be used to assess the bivariate effects of the interventions and to analyze within-group and between-group differences and changes. In addition, logistic regression models and/or cluster analyses will be performed to analyze issues related to dose–response and responder characteristics. SPSS 22 (SPSS Inc., Chicago, IL, USA) will be used for the statistical analysis.

### Limitations

Individuals with pre-diabetes and diabetes are randomized in the same group and it can be discussed whether the same decline in HbA_1c_ can be expected in individuals with pre-diabetes. Furthermore the participants in the control group may be influenced by participating in a research study and being assessed regularly. As they agree to join they are highly motivated to change their physical activity level and we expect many of them to succeed in this by their own. This could make comparison of between-group differences problematic. Moreover, the control group is assessed by the same staff as group A and B, for practical and financial reasons. This staff is trained in motivational interviewing and it might be difficult to treat the patients differently. At measurements participants from group A, B and C are given different schedules, group A participants are offered more time and a systematic motivational interviewing following a set of questions.

## Discussion

With an ageing population and increasing incidence of pre-diabetes and diabetes, it is of high importance to construct, implement and evaluate cost-effective preventive methods adjusted for the health care system [41]. It is well-documented that walking, exercise groups and advice on prescription are effective at a low cost per participant over 12 months [34]. It is known that more support gives more long-term effect, although it is also evident that rather simple interventions can provide a worthwhile effect [67]. This study aims at bringing more knowledge to what intensity such methods need to be at, to be as cost-effective as possible. Long-term interventions are rare in diabetes intervention research [41]. This study will show if support for two year’s maintenance is effective. A further strength is that it brings theory into practice and adds evidence for methods by which behavior change techniques may be delivered and embedded in the day to day care in a time-effective way [43, 68]. The methods used and evaluated in Sophia Step Study may be easily implemented in the primary health care setting with little new competence and administrative time needed. The study aims to raise the patient’s awareness and self-management and assess the impact of self-perceived general health, as proposed by the US National Standards for Diabetes Self-Management Education and Support [69], WHO Europe’s policy framework Health 2020 [26] and value-based care [70] which is being implemented in Sweden.

### Ethical considerations

Ethical approval was obtained from the ethics committee of the Regional Ethical Review Board (Dnr.2012/1570-31/3). All participants receive both oral and written information and are asked to give written informed consent prior to participating. The participants are informed that the data is treated confidently. All data will be stored anonymous with only the ID-code and analysis will be made only at group level. The study will be carried out in accordance with the ethical principles of the World Medical Association Declaration of Helsinki: ethical principles for medical research involving human subjects (2013).
